# Identification of super-enhancer-based biomarkers for predicting survival and immunotherapy efficacy in colorectal cancer

**DOI:** 10.7150/jca.119265

**Published:** 2026-01-01

**Authors:** Yanan Yu, Xiuxiu Zhang, Xiaolin Ma, Jiao Ren, Jinglei Zhang, Luoyu Zhu, Yanfang Chen, Zhong Lu, Jiaqiu Li

**Affiliations:** 1 Department of Oncology, Affiliated Hospital of Shandong Second Medical University, School of Clinical Medicine, Shandong Second Medical University, Weifang 261053, Shandong, China.; 2 Department of Medical Oncology, Rizhao central hospital, Rizhao 276801, Shandong, China.; 3 Department of Radiotherapy, Affiliated Hospital of Shandong Second Medical University, Shandong Second Medical University, Weifang 261053, Shandong, China.

**Keywords:** colorectal cancer, super-enhancer, tumor immunity, immune checkpoint inhibitors, biomarkers, bioinformatics

## Abstract

Immune checkpoint inhibitors are effective treatments for many tumors. However, existing biomarkers can benefit only a small selection of colorectal cancer patients. Super-enhancers are associated with various tumor characteristics. We wondered whether super-enhancer-related genes could be novel biomarkers for immunotherapy. We screened super-enhancer-related genes that were highly correlated with immune infiltration through weighted gene co-expression network analysis on the basis of chromatin immunoprecipitation sequencing data. A prognostic risk signature was established using least absolute shrinkage and selection operator and cox regression models. By analyzing the correlations between the expression of model genes and the immunophenotypic and microsatellite instability scores, we determined that *PLAU* and *GSDMC* expression had high predictive value for immunotherapy efficacy. Moreover, we predicted the sensitivity of the PLAU and GSDMC proteins to drugs by virtual docking. Finally, we validated the effect of the super-enhancer activity on *PLAU* and* GSDMC* expression. Overall, our study identified super-enhancer-based biomarkers for predicting survival and immunotherapy efficacy in colorectal cancer.

## Introduction

According to the report from the American Cancer Society in 2023 1, colorectal cancer (CRC) is the third most common cancer in the world in terms of incidence and mortality. Despite the popularization and development of radiotherapy, chemotherapy, and surgery for cancer treatment 2-4, CRC continues to have a high mortality around the world, and that rate is only increasing. A poor prognosis is the principal cause of the high mortality of CRC 5. The development of immunotherapy has provided a new option for the treatment of CRC 6, and immunotherapy alone or in combination with traditional therapy has become a cornerstone of CRC treatment in clinical practice 7, 8. The tumor microenvironment (TME) is composed of stromal cells, immune cells, vascular endothelium, and intravascular blood cells 9. Among them, immune cells play dual roles in antitumor activity and tumor immune escape 10, 11. Immunotherapy achieves the therapeutic effect of controlling or eliminating tumors by externally interfering with the body's immune system to restart the "tumor-immunity" cycle of recognizing and killing tumor cells 12. Immunotherapy is superior to traditional treatments because of its high specificity and less side effects 13, 14.

Currently, inhibitors targeting immune checkpoint proteins such as PD-1, PD-L1, and CTLA-4 have been broadly applied in clinical practice 12, 15. However, considering some specific side effects, such as peripheral neuropathy, headache 16 and hearing loss 17, caused by immune checkpoint inhibitors (ICIs), it needs to exactly select the patients suitable for ICIs. Some biomarkers have been identified and are considered predictive factors for CRC immunotherapy response, such as the immunophenotype score (IPS) 18, microsatellite instability (MSI) 19, and tumor mutation burden (TMB) 20, 21, but the currently known biomarkers can only recommend immunotherapy to be administered to a small percentage of patients, and the efficacy of immunotherapy on these biomarker-indicated patients is also not reliable. Identifying specific biomarkers is the way forward for tumor immunotherapy, allowing it to benefit more patients 22. In recent years, new biomarkers for predicting the response of tumors treated with ICIs have been widely studied. For example, studies have shown that the Royal Marsden Hospital (RMH) score is an independent biomarker for predicting PFS in NSCLC patients receiving ICI treatment 23.

A super-enhancer (SE) is a large cluster of multiple adjacent common enhancers. Super-enhancers drive the regulation of cell fate-related gene expression. Specific histone modifications, particularly histone 3 lysine 27 acetylation (H3K27ac), are essential for super-enhancer activity 24. As powerful transcription regulators, super-enhancers play crucial roles in the accumulation of many major transcription factors and other cofactors 25, 26. Bromodomain and outer end (BET) proteins, such as BRD4, recognize and bind to histone acetylated lysine residues in the super-enhancer region to facilitate gene transcription 27. Studies have shown that immune evasion in CRC is also regulated by super-enhancers 28. Thus, we wondered whether these genes regulated by super-enhancers could serve as new biomarkers for predicting the efficacy of immunotherapy. Inhibitors or drugs that target these genes may yield improved outcomes in immunotherapy. Given the important roles of super-enhancers in cancer, this study aimed to identify new super-enhancer-related biomarkers that can predict the immunotherapy efficacy and outcomes of patients with colorectal cancer.

## Materials and Methods

### Screening super-enhancer-related genes on the basis of H3K27ac ChIP-seq data

The ChIP-seq data have been deposited in the Gene Expression Omnibus (GEO) under accession number GSE198223. The sequencing data were aligned to the human HG19 genome using BOWTIE V2.1.0 software. The data were transferred for peak calling by MACS V1.4.2 software. Statistically significant ChIP-enriched peaks were identified by comparison of the IP data with the input data. An enrichment region was regarded as a region containing constituent enhancer lines and was used to calculate the identity signal. The ROSE tool was used to generate a stitched enhancer by combining the enhancers within a range of 12.5 kb. All stitched enhancers were ranked by the signal of the IP group after subtracting the input group, and super-enhancers were identified based off of a specific threshold for the stitched enhancer signal. The super-enhancers were annotated by the correlated coding genes using the newest Arraystar UCSC database and other UCSC RefSeq databases.

### Weighted gene co-expression network analysis (WGCNA)

First, we utilized the R package "ESTIMATE" to calculate the immune score, stromal score, estimated score, and tumor purity for the samples in the TCGA-COAD (colon cancer) cohort 29. After conducting WGCNA to cluster the samples, we set the cutHeight to 4000 based on the degree of sample dispersion and used the 'cutreeStatic' function to remove 10 samples with a height above 4000 for data cleaning. In the end, we included a total of 455 patients with COAD in this study. We used a topological overlap matrix to determine the appropriate soft threshold parameter to use (β=5). Next, we used dynamic hybridization cleavage to identify co-expressed gene modules. Hierarchical clustering trees were created using dynamic hybrid cutting, where genes with similar functions or expression data form a branch, which in turn forms a gene module. Finally, we calculated the Pearson correlation between modular signature genes (MEs) and the immune infiltration score, as well as tumor purity. A significant correlation was considered when adjust* P* < 0.05. Using normalized TCGA-COAD expression profile data and the CIBERSORT algorithm, an LM22 gene matrix file containing 22 immune cell subtypes was used to calculate the proportions of 22 immune cell types. The composition of the immune cells was visualized using a box plot.

### Dataset and sample extraction

We acquired RNA sequencing expression data, genomic mutation data and associated clinical data for COAD patients from the TCGA database. The count data were analyzed using the R package "DESeq2", and the differential genes were selected on the basis of a |log_2_fold change|≥1 and adjust* P* < 0.05. Normalized FPKM data were used to generate heatmaps of differentially expressed genes using the R package "pheatmap". The volcano plot was visualized using the packages "ggpubr" and "ggthemes".

### Prognostic analysis

Using the TIDE database 30 for prognostic analysis of the GEO CRC datasets, we analyzed the correlation between gene expression and overall survival (OS) and disease-free survival (DFS) with a cutoff p-values of 0.05. The “Query gene” module in the TIDE database was used to analyze the 9 immune infiltration-related differentially expressed genes. We obtained their prognostic value in multiple CRC datasets in the “Expression” section. The optimal critical value was calculated via the "surviviner" software package, and the survival curves of the six target genes were validated through TPM data in the TCGA-COAD queue.

### Construction of the prognostic risk model

On the basis of 404 samples with clinical information from the TCGA-COAD cohort, the LASSO-Cox model (R package "glmnet") identified 6 risk genes related to immune infiltration in the training group. The risk score based on this model was subsequently calculated. The R package "survminer" was used to determine the optimal cutoff point for the high- and low-risk groups. Next, we performed survival analysis and plotted Kaplan‒Meier (K‒M) curves with the "survival" and "survminer" packages. Finally, using the 'time-ROC' package, we plotted ROC curves to calculate the 1-, 3-, and 5-year survival rates of patients. The prognostic model was subsequently validated with a validation set.

### Immunotherapy efficacy prediction

In 2017, Charoentong et al. 31 reported that the IPS could predict the response to immune checkpoint inhibitors. Therefore, we downloaded the IPS of TCGA-COAD patients from the TCIA database, tested the relationship between gene expression levels and the IPS with the Wilcoxon rank sum test, and visualized it using the "ggpubr" package. In addition, previous research has shown that patients with MSI-H tumors have a better response to ICIs 20, 32. Consequently, using the TCGA module in the integrated bioinformatics analysis platform ACLBI, we analyzed the correlation between single gene expression and the MSI score in COAD to estimate the predictive effect of single gene expression on the efficacy of ICIs. The "Biomarker Evaluation" module in the TIDE database was used to compare the target gene with other biomarkers. The predictive performance of biomarkers for the treatment effect of different cohorts of ICIs was expressed by the AUC. The larger the AUC was, the better the predictive effect. We subsequently analyzed the correlation between target gene expression and cytotoxic T cell (CTL) function by the 'Query Gene' module. The relationships between the expression levels of *PLAU* and *GSDMC* in melanoma and the outcomes of patients receiving immunotherapy were validated through the 'immunotherapy' module in the Kaplan‒Meier plotter database.

### Correlation between gene expression and the tumor microenvironment

Sangerbox 3.0 was used to analyze the relationship between gene expression and the tumor microenvironment. First, we analyzed the data via immunoinfiltration analysis in the "Pan-cancer analysis" module. The data were obtained from "TCGA+GTEx" and subjected to log (x+0.001) transformation. In the immune cell analysis (TIMER) module, we subsequently assessed the Spearman rank correlation between gene expression and immune cells in the tumor microenvironment. Finally, we assessed the relationships between gene expression and immune checkpoint genes through the ACLBI website.

### Drug sensitivity analysis and molecular docking

We downloaded the IC50s (half-maximal inhibitory concentrations) of the drugs and gene expression levels of colorectal cancer cell lines from the GDSC2 and CTRPv2 databases. The correlation of *PLAU* and *GSDMC* expression with the drug IC50 was calculated using R software, and scatter plots were generated. The 3D structures of the proteins were obtained from the Protein Data Bank (PDB) database. The structures of the small molecule drugs were downloaded from the PubChem database. The binding sites of the proteins were identified by the GHECOM algorithm. AutoDock Tools was used for the molecular docking of proteins to small molecule drugs. Then, we visualized the docking results via PyMOL software. Finally, Ligplus software was used to determine the two-dimensional structure of protein amino acid residues that interact with small-molecule drugs.

### Cell culture

The human colon cancer cell lines HCT116 and DLD1 were used for this study. The cells were obtained from the Cell Bank of the Chinese Academy of Sciences. HCT116 and DLD1 cells were cultured at 37 °C in a 5% CO_2_ incubator in McCoy's 5A medium or RPMI 1640 medium supplemented with 10% fetal bovine serum. The cells were tested for mycoplasma contamination every two months.

### Quantitative real-time PCR (qRT‒PCR)

The cells in the 6-well plate were treated with the BRD4 inhibitors-JQ1 and I-BET-762 for 24 hours, after which the RNA was extracted from the cells via TRIzol. The qPCR detection method was the same as previously reported 33. β-actin was used as the endogenous control for normalization. The sequences of the primers used are as follows:

*PLAU*-F: CAGATTCCTGCCAGGGAGAC, R: GCCAGGCCATTCTCTTCCTT; *GSDMC*-F: AGGTCATTTGGATGGCCCTG, R: CCAGGATGCTCCTTACCAGC; *β-actin*-F: CACCAACTGGGACGACAT, R: ACAGCCTGGATAGCAACG.

### Chromatin immunoprecipitation-qPCR

ChIP assays were conducted as reported in previous studies 34 with the anti-H3K27ac (Abcam, ab177178, Cambridge, UK), anti-P300 (Abcam, ab275378, Cambridge, UK) and anti-BRD4 (Bethyl Laboratories, A301-985A100, Montgomery, TX, USA) antibodies. The primer sequences for the *PLAU* and *GSDMC* super-enhancer regions are shown below:

*PLAU*-E1-F: GCAAGGCACCCTCGTACTTT, *PLAU*-E1-R: GCAAGTTCACGCTTAGACAGC; *PLAU*-E2-F: AGACTCACCCTGTGCCTACA, *PLAU*-E2-R: CCCGGAGGCAACCCAATAAT; *PLAU*-E3-F: TGTTGAAGAGTCCGTCTGCC, *PLAU*-E3-R: TTGTCTGTGTCCCTGTGTGG; *PLAU*-Promoter-F: GGTGTCACGCTTCATAACGG, *PLAU*-Promoter-R: GGCTGTCATGCTGATTGCTG; GSDMC-E1-F: ACCTCCTGCGGTCAAGTAGA, GSDMC-E1-R: AGTCAGGGACCCATTGGTGA.

### H3K27ac ChIP-seq tracking

We downloaded H3K27ac ChIP-seq data for cell lines from the ENCODE database 35. The WashU Epigenome Browser was used to visualize the H3K27ac tracks.

### Electrophoretic mobility shift assay (EMSA) and super-shift EMSA

First, we extracted the nuclear proteins from HCT116 cells via the Nuclear and Cytoplasmic Protein Extraction Kit (Beyotime, P0028) according to the protocol described in the user guide. After BCA quantification, the final concentration of protein was adjusted to 1 µg/µl. The proteins were subsequently mixed with the probes and incubated at room temperature for 30 minutes. Two microliters of sample mixture mixed with 4 µl of loading dye (6×) was loaded into each gel lane. The gel was run in 0.5×TBE buffer at 150 V for 60 min and transferred to a nylon membrane at 300 mA for 30 min. Then, the membrane was crosslinked for 20 min with an ultraviolet lamp and sealed for 20 min. The membrane was incubated with diluted antibodies for 30 min at room temperature. Finally, after the membrane was eluted and dried, graphs were obtained using a chemiluminescence apparatus. The probes were synthesized by Servicebio (Wuhan, China). The probe sequences are listed below:

E1: F: 5'-Biotin-GCAAGGCACCCTCGTACTTT-3', R: 5'-Biotin-AAAGTACGAGGGTGCCTTGC-3'; E1: Cold probe F: 5'-GCAAGGCACCCTCGTACTTT-3', R: 5'-AAAGTACGAGGGTGCCTTGC-3'; E1: Mutant probe F: 5'-GCAATACAGACTCCTAGCAG-3', R: 5'-CTGCTAGGAGTCTGTATTGC-3'; E2: F: 5'-Biotin-AGACTCACCCTGTGCCTACA-3', R: 5'-Biotin-TGTAGGCACAGGGTGAGTCT-3'; E2: Cold probe F: 5'-AGACTCACCCTGTGCCTACA-3', R: 5'-TGTAGGCACAGGGTGAGTCT-3'; E2: Mutant probe F: 5'-AGACGAACCTTCCGCCTATT-3', R: 5'-AATAGGCGGAAGGTTCGTCT-3'; E3: F: 5'-Biotin-TGTTGAAGAGTCCGTCTGCC-3', R: 5'-Biotin-GGCAGACGGACTCTTCAACA-3'; E3: Cold probe F: 5'-TGTTGAAGAGTCCGTCTGCC-3', R: 5'-GGCAGACGGACTCTTCAACA-3'; E3: Mutant probe F: 5'-TGTGGAATACTTCGTCTGCC-3', R: 5'-GGCAGACGAAGTATTCCACA-3'.

### Tissue samples collection and analysis

This research was approved by The Institute of Research Medical Ethics Committee of the Affiliated Hospital of Shandong Second Medical University. Colorectal cancer tissues and paired normal tissues from eighteen patients were collected from the Affiliated Hospital of Shandong Second Medical University. All patients signed the informed consent forms. The RNA extraction and qPCR detection method is the same as above.

### Statistical analysis

Statistical significance was assessed with a two-sided Student's t test via GraphPad Prism 7 software. The results are expressed as the means ± standard deviations. If the results did not have the same standard deviation, a t test with Welch's correction was used. In addition, statistical tests were performed using R software version 4.2.2, and p-values less than 0.05 were considered to indicate statistically significant differences.

## Results

### Screening super-enhancer-related genes correlated with immune infiltration through WGCNA

**Figure 1:**
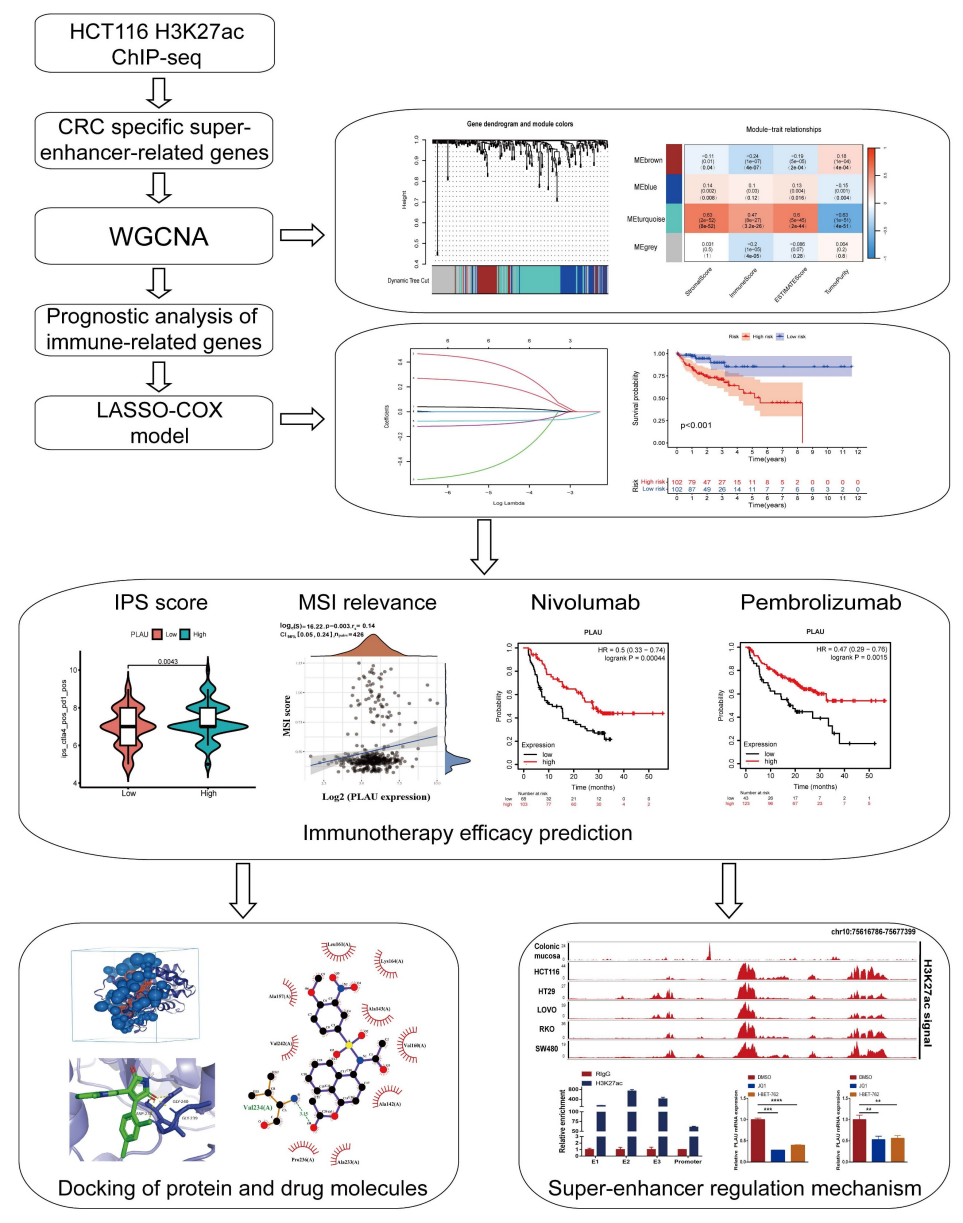
Workflow of identification of CRC specific super-enhancer-related genes for immunotherapy efficacy prediction. CRC, colorectal cancer; WGCNA, weighted gene co-expression network analysis; LASSO, least absolute shrinkage and selection operator; IPS, immunophenotypic score; MSI, microsatellite instability.

**Figure 2:**
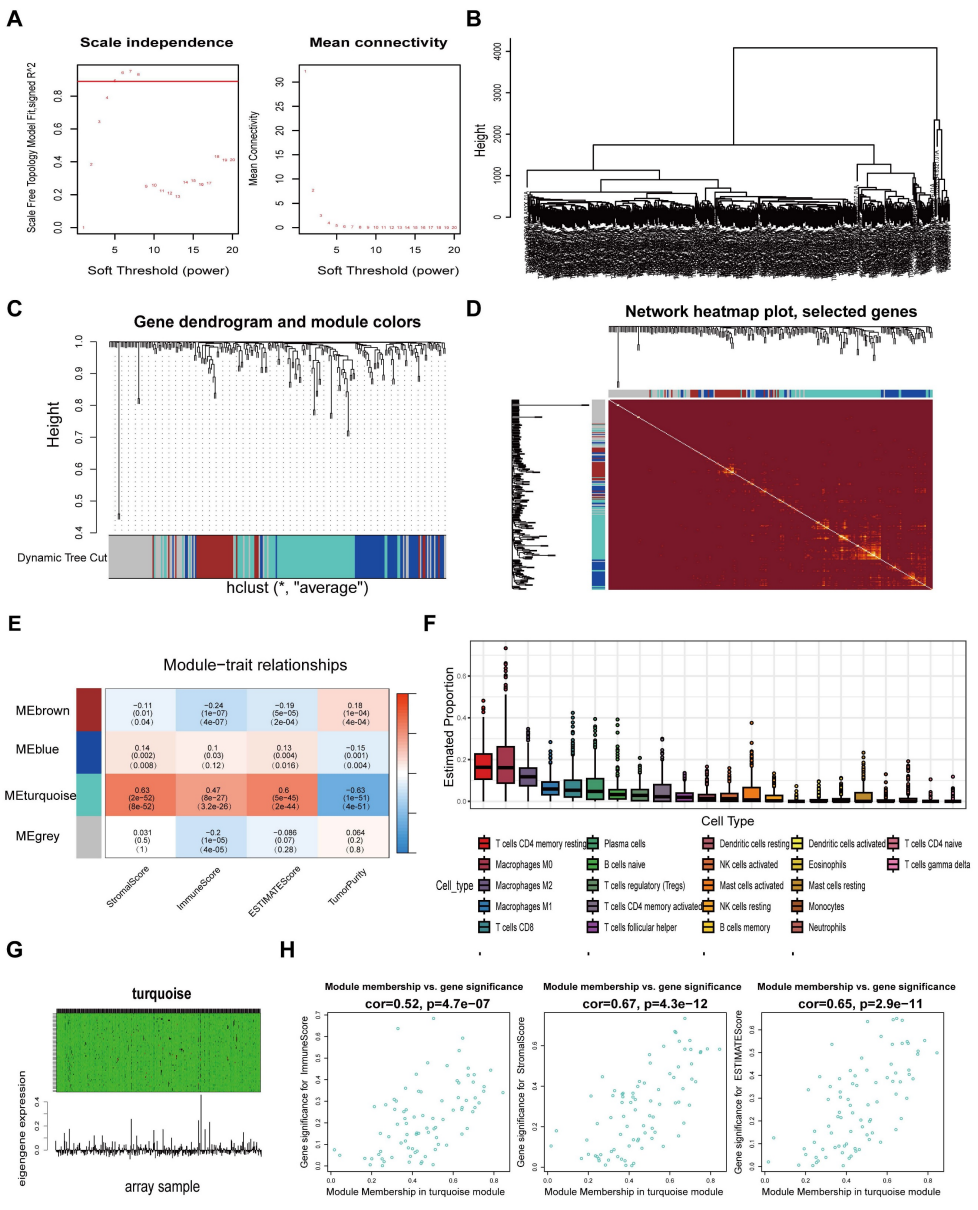
Screening super-enhancer-related genes correlated with immune infiltration through weighted gene co-expression network analysis (WGCNA). **A** Scale indpendence and Mean connectivity for Multiple Soft Thresholds. **B** Sample Clustering Tree. **C** Clusters and merging of gene co-expression modules. **D** Heatmap of gene co-expression networks. **E** Correlation and p-values of immune infiltration and tumor purity with co-expression modules. **F** Determine the proportion of various immune cells through the Cibersort algorithm. **G** Heatmap of the association between gene expression and the turquoise module. **H** Scatter-plots of the correlation between the turquoise module and the immune infiltration scores.

A schematic flow chart diagram of this study is shown in Fig. 1. In our latest study 36, we obtained 383 super-enhancer-related mRNAs via ChIP-seq with the anti-H3K27ac antibody in HCT116 cells (GSE198223). To further identify colorectal cancer-specific super-enhancers, we downloaded and analyzed the H3K27ac signal of these 383 genes in normal colonic mucosa by the ChIP-seq data from the ENCODE database. We compared their H3K27ac signal in the HCT116 cell and normal colonic mucosa. If their H3K27ac signal in the HCT116 cell was higher than that in normal colonic mucosa, we regard them as specific super-enhancers in the HCT116 cell line. Ultimately, we identified 239 CRC-specific super-enhancer-related genes. To assess the correlation between these genes and the tumor immune microenvironment, we conducted calculations for the immune score, stromal score, estimate score, and tumor purity using the TCGA-COAD datasets. A total of 455 COAD patients were selected from the TCGA database for weighted gene co-expression network analysis (WGCNA). The scale-free fit indices at different soft thresholds (powers) are shown in Fig. 2A. We determined β = 5 to be the optimal power value. Moreover, we performed a sample clustering analysis (Fig. 2B). After the samples were clustered, the adjacent matrix was transformed to create a TOM matrix, and the function tree generated by hierarchical clustering was examined for modules. The dynamic tree cutting method was used to merge modules with values greater than 0.75, resulting in a module clustering tree plot (Fig. 2C) and the construction of mRNA co-expression networks (Fig. 2D). We performed a correlation analysis of the modules and trait groupings generated by the clustering. We created a heatmap of the modules and trait data, as shown in Fig. 2E. The correlations and adjust p-values between each module and trait are presented in the heatmap. Among them, the turquoise module has the highest correlation.

We subsequently calculated the proportion of immune cells in the immune microenvironment of patients with COAD via the CIBERSORT algorithm (Fig. 2F). We found that resting CD4 memory T cells accounted for the largest percentage of immune microenvironment immune cells in the COAD cohort. Previous studies have shown that the subgroups with more resting CD4 memory T cells have reduced immune escape and the patients will have a better clinical outcome 37. Afterwards, we constructed a composite chart to display the expression characteristics of turquoise module genes and module eigengenes (MEs) (Fig. 2G). The scatter plot results indicated that the correlations between the turquoise module genes and immune infiltration scores (immune score, stromal score and estimate score) were 0.52, 0.67, and 0.65, respectively (Fig. 2H). These results suggest that the genes in the turquoise module are significantly positively correlated with the immune infiltration scores. Because immune cells are crucial in combating tumor immune responses, further research on the 83 genes in this module may help to identify biomarkers and new therapeutic targets for immunotherapy in colorectal cancer.

### Differential gene and prognostic analysis

**Figure 3:**
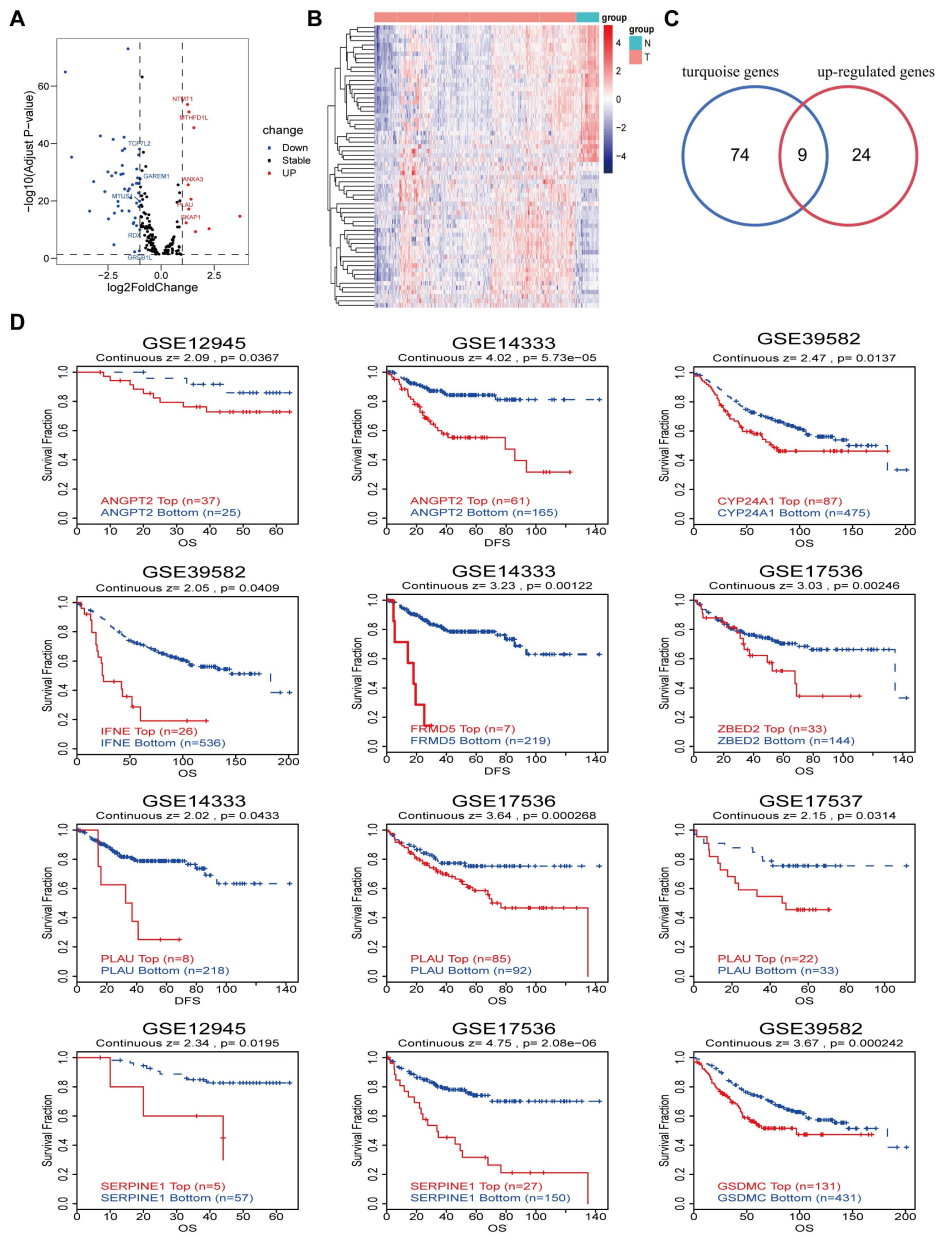
Differential genes and prognostic analysis.** A-B** Volcano map and heatmap of differential genes based on the TCGA-COAD datasets. **C** Venn diagram of the intersection of up-regulated genes and turquoise module genes. **D** Kaplan-Meier curves show the prognostic value of the intersection genes in CRC using TIDE database.

On the basis of the TCGA-COAD datasets, we analyzed the differential expression of genes associated with super-enhancers, resulting in the generation of a volcano plot and heatmap (Fig. 3A-3B). This analysis revealed a total of 33 upregulated genes and 32 downregulated genes. Overlaying the upregulated genes with the 83 immune infiltration-related genes in the above turquoise module via Venn diagrams yielded 9 immune infiltration-related differentially expressed genes (Fig. 3C). Moreover, prognostic analysis of the aforementioned 9 genes was conducted using the GEO datasets obtained from the TIDE database. The results of the K‒M curve analysis revealed that CRC patients with elevated expression levels of 8 specific genes (*ANGPT2, CYP24A1, IFNE, PLAU, FRMD5, SERPINE1, ZBED2,* and *GSDMC*) had unfavorable prognoses (Fig. 3D). In addition, we further validated the relationship between the expression of 6 target genes in the TCGA-COAD cohort and the outcomes of cancer patients by determining the optimal threshold (Fig. S1A and Table S1).

### Construction of the prognostic risk model

**Figure 4:**
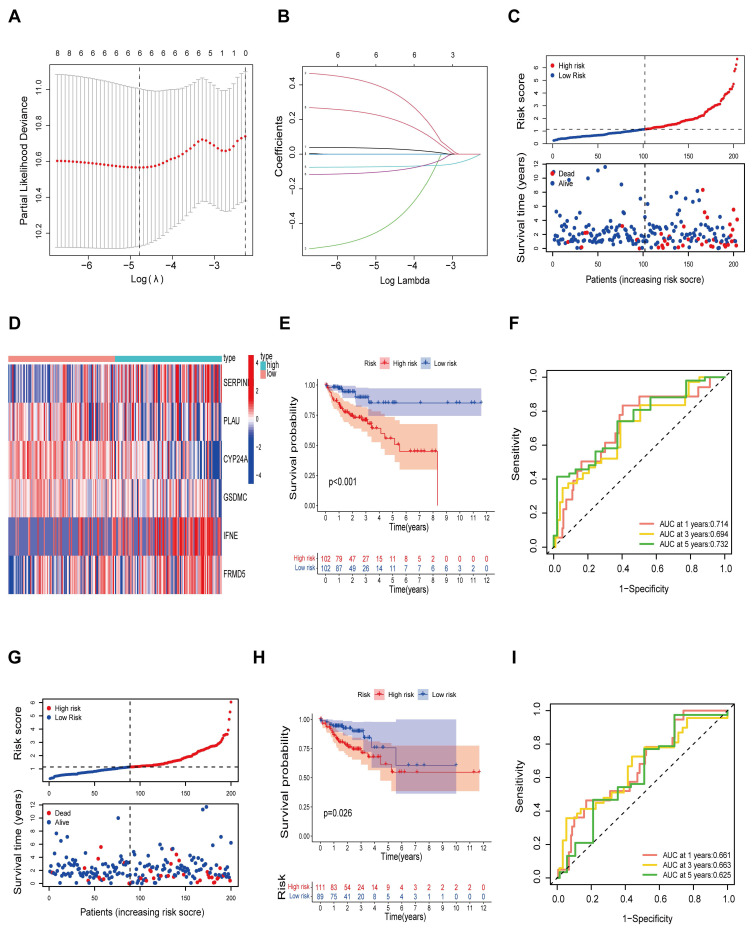
Construction of prognostic risk model. **A** Screening for turning to optimal parameters (lambda) in the LASSO model. **B** LASSO coefficient curve for prognostic genes. **C** Distribution of risk score and survival time of the high and low risk groups. **D** Heatmap of six candidate genes expression in the high and low risk groups. **E** Kaplan-Meier curves for overall survival of patients in the high and low risk groups. **F** Time-ROC curves of the overall survival at 1, 3 and 5 years. **G** Distribution of risk score and survival time of the high and low risk groups in the test cohort. **H** Kaplan-Meier curves for overall survival of patients in the high and low risk groups in the test cohort. **I** Time-ROC curves of the overall survival at 1, 3 and 5 years in the test cohort.

Next, we further explored and validated the prognostic value of the 8 prognosis-related genes through TCGA data. A total of 404 patients were recruited to participate in the study by removing some samples with a total survival time of 0 and incomplete clinicopathological information from the TCGA-COAD dataset. We subsequently randomly divided 404 patients into a training set (n = 203) and a validation set (n = 201) at a 1:1 ratio. We identified 6 risk genes through LASSO-Cox regression analysis (Table S2) and constructed immune infiltration risk features (Fig. 4A-B). We calculated the risk score on the basis of the expression levels of the 6 risk genes via the following formula: risk score=FRMD5*(0.2161)+SERPINE1*(0.3810)+IFNE*(0.0325)-CYP24A1*(0.0722)-GSDMC*(0.1007)-PLAU*(0.4265). Patients in the training set were divided into a high-risk group (n = 101) and a low-risk group (n = 101) according to the risk score. We observed that as the risk score increased, the mortality of the patients increased (Fig. 4C). In addition, we constructed a heatmap to display the expression levels of the 6 risk genes in the high- and low-risk groups (Fig. 4D). We subsequently conducted K‒M survival analysis (Fig. 4E) for patients in the high- and low-risk groups, which revealed that patients in the high-risk group had markedly shorter survival times than did those in the low-risk group (p < 0.01). Time‒ROC analysis revealed that the AUCs for survival at years 1, 3 and 5 were 0.714, 0.694 and 0.732, respectively, indicating the good sensitivity and specificity of the prognostic model developed for the 6 risk genes in predicting the survival of patients with COAD (Fig. 4F). We then validated the prognostic signature of the 6 risk genes in the test cohort. Similarly, survival analysis of the test cohort revealed significant differences in survival time between the test groups, with significantly shorter OS in the high-risk group (P < 0.05) (Fig. 4G-H). In addition, analysis of the time-ROC results for the test cohort revealed AUCs of 0.661, 0.663, and 0.625 at years 1, 3 and 5, respectively (Fig. 4I).

### PLAU and GSDMC as biomarkers to predict immunotherapy efficacy

**Figure 5:**
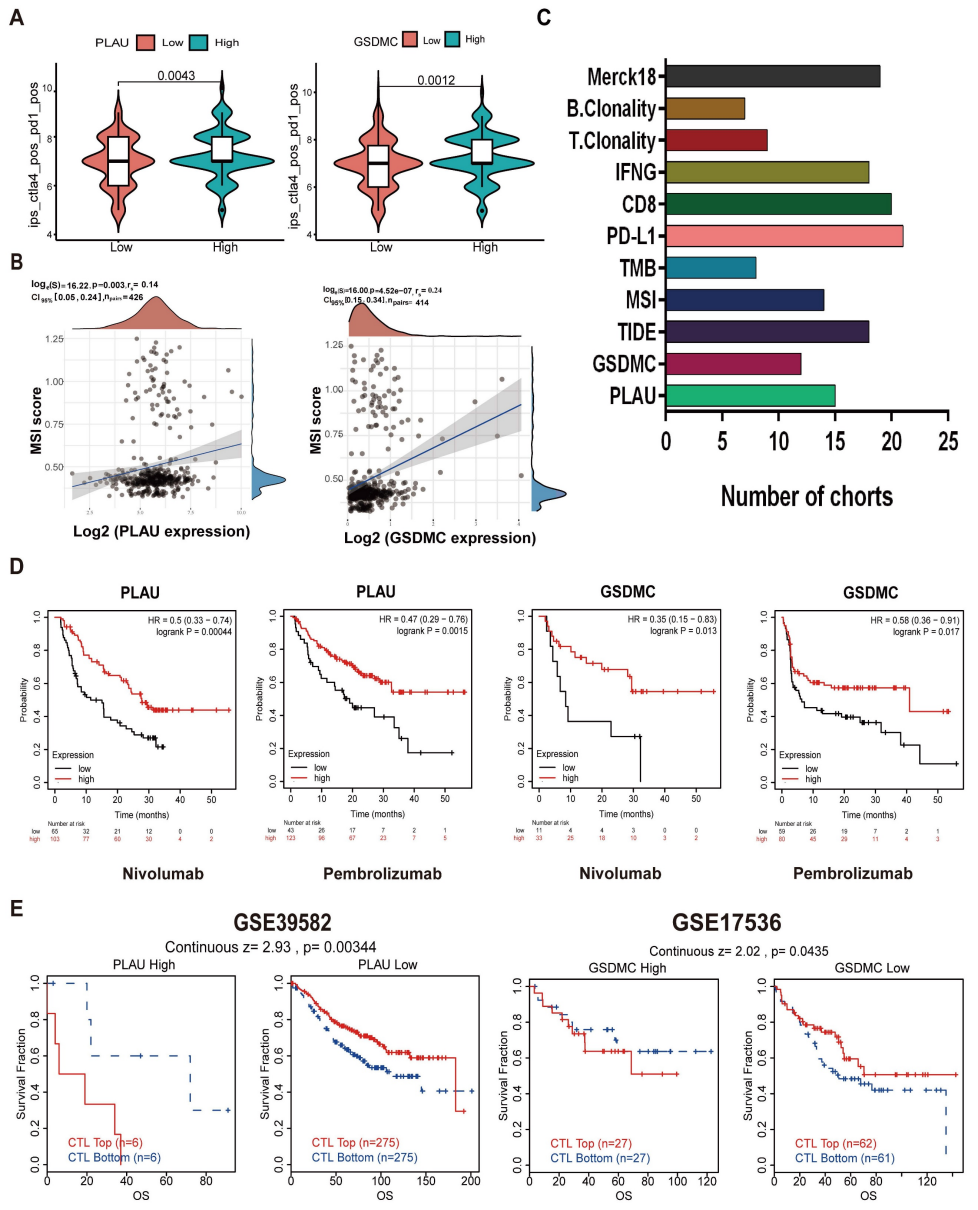
*PLAU and GSDMC* as biomarkers to predict immunotherapy efficacy. **A** Relationship between *PLAU/GSDMC* expression and IPS scores. **B** Correlation between *PLAU/GSDMC* expression and MSI scores. **C** The predictive capability of biomarkers for immunotherapy efficacy in multiple human immunotherapy cohorts. **D** Relationship between high and low *PLAU, GSDMC* expression and prognosis in melanoma patients when treated with PD-1 inhibitors. **E** Overall survival of colorectal cancer patients with different *PLAU/GSDMC* expression and Cytotoxic T cell (CTL) levels.

We then wanted to validate our above hypothesis by revealing the relationship between gene expression levels and immunotherapy efficacy. The patient's antitumor immune response can be determined by the immunophenotypic score (IPS). We downloaded the TCGA-COAD IPS score data from the TCIA database and analyzed the expression of each of the 6 risk genes in relation to the IPS score. Among them, the expression of *PLAU* and *GSDMC* was significantly correlated with the IPS score. As depicted in Fig. 5A and Fig S1B, immunotherapy was more effective in the high *PLAU* or high *GSDMC* expression group when combined with CTLA4 inhibitors and PD-1 inhibitors. MSI is closely related to the antitumor immune response and is an important biomarker for assessing the efficacy of immunotherapy. Our results revealed a positive correlation between the expression of *PLAU/GSDMC* in CRC and the MSI score (Fig. 5B). *GSDMC* (r = 0.24, *p* < 0.0001) has a higher correlation with MSI score than *PLAU* (r = 0.14, *p* < 0.01). Next, we compared the predictive capability of *PLAU/GSDMC* for immunotherapy efficacy in multiple human immunotherapy cohorts (Fig. S2-S3). AUC values > 0.5 means that the algorithm outperformed random. In Fig. 5C, we compiled histograms for the cohorts with an AUC > 0.5 and found that *PLAU* had better predictive value than existing immunotherapy efficacy biomarkers, such as MSI and TMB. *GSDMC* also had a greater predictive value than TMB. In particular, we found that melanoma patients with high expression of *PLAU* or *GSDMC* had better prognosis when treated with PD-1 inhibitors (nivolumab and pembrolizumab) (Fig. 5D). In short, the above data showed that *PLAU* and *GSDMC* could be used as new biomarkers to predict the immunotherapy efficacy. In addition, we found that the function of cytotoxic T-cell (CTL) may be inhibited by high expression of *PLAU/GSDMC* (Fig. 5E). Because CTL is the effector cell responsible for immune response, we assumed that high expression of *PLAU/GSDMC* may affect the antitumor immune response of CTLs. This result implied that *PLAU/GSDMC* may also be new targets for immunotherapy.

### Correlation between *PLAU/GSDMC* and the tumor microenvironment (TME)

We utilized the estimation algorithm to evaluate the expression status of *PLAU* and *GSDMC* in the TME. We analyzed the Spearman correlations between the expression of these two genes and the matrix score, immune score, and estimate score in the tumor microenvironment using Sangerbox 3.0. The results revealed a positive correlation between *PLAU/GSDMC* expression and the immune infiltration score (Fig. S4A). In addition, we investigated the potential correlation between the expression of *PLAU*/*GSDMC* and tumor-associated immune cells. As shown in Fig. S4B, the expression of *PLAU*/*GSDMC* was positively correlated with the abundances of B cells, CD4+ T cells, CD8+ T cells, neutrophils, macrophages, and dendritic cells. We further analyzed the correlation between *PLAU*/*GSDMC* expression and PD-1, PD-L1, and CTLA4 expression in COAD using the ACLBI database. The scatter plots revealed a positive correlation between the expression of *PLAU*/*GSDMC* in COAD and that of immune checkpoint genes (Fig. S4C). Overall, the expression of *PLAU* and *GSDMC* was positively correlated with the TME, indicating their potential as new targets for immunotherapy in CRC.

### Predicting the sensitivity of drugs for PLAU and GSDMC proteins

**Figure 6:**
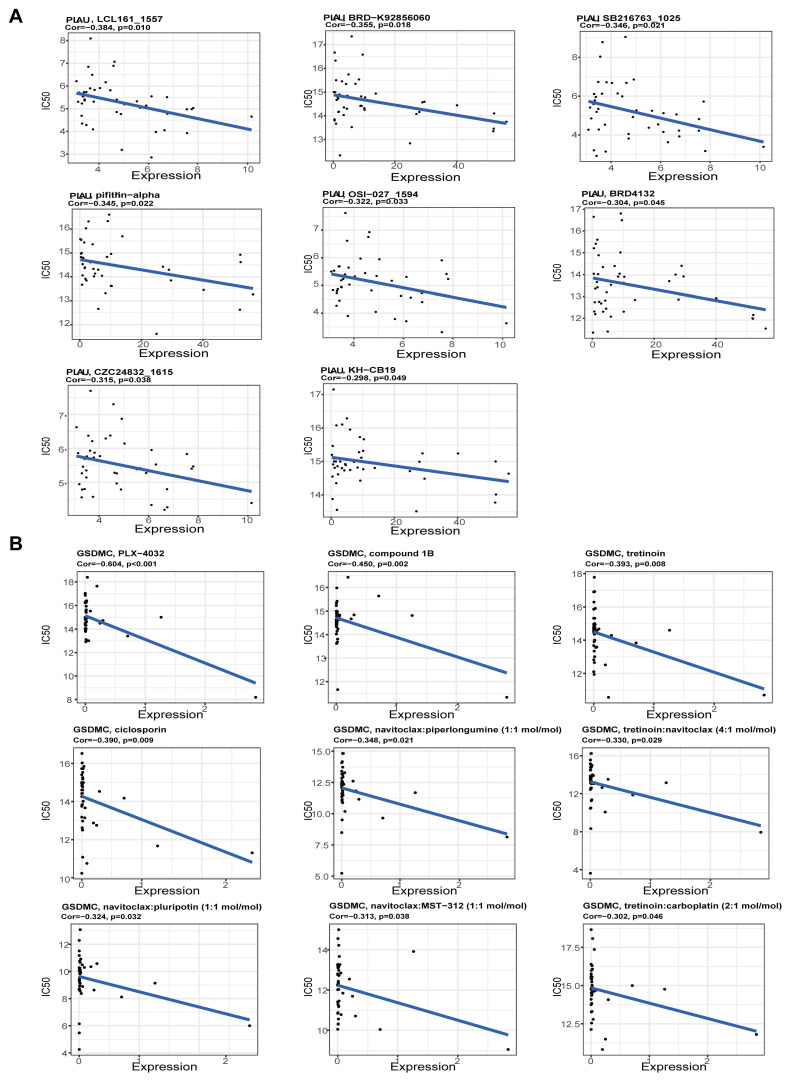
Scatter plots of the correlation between *PLAU* (A), *GSDMC* (B) expression and the IC50 of drugs.

**Figure 7:**
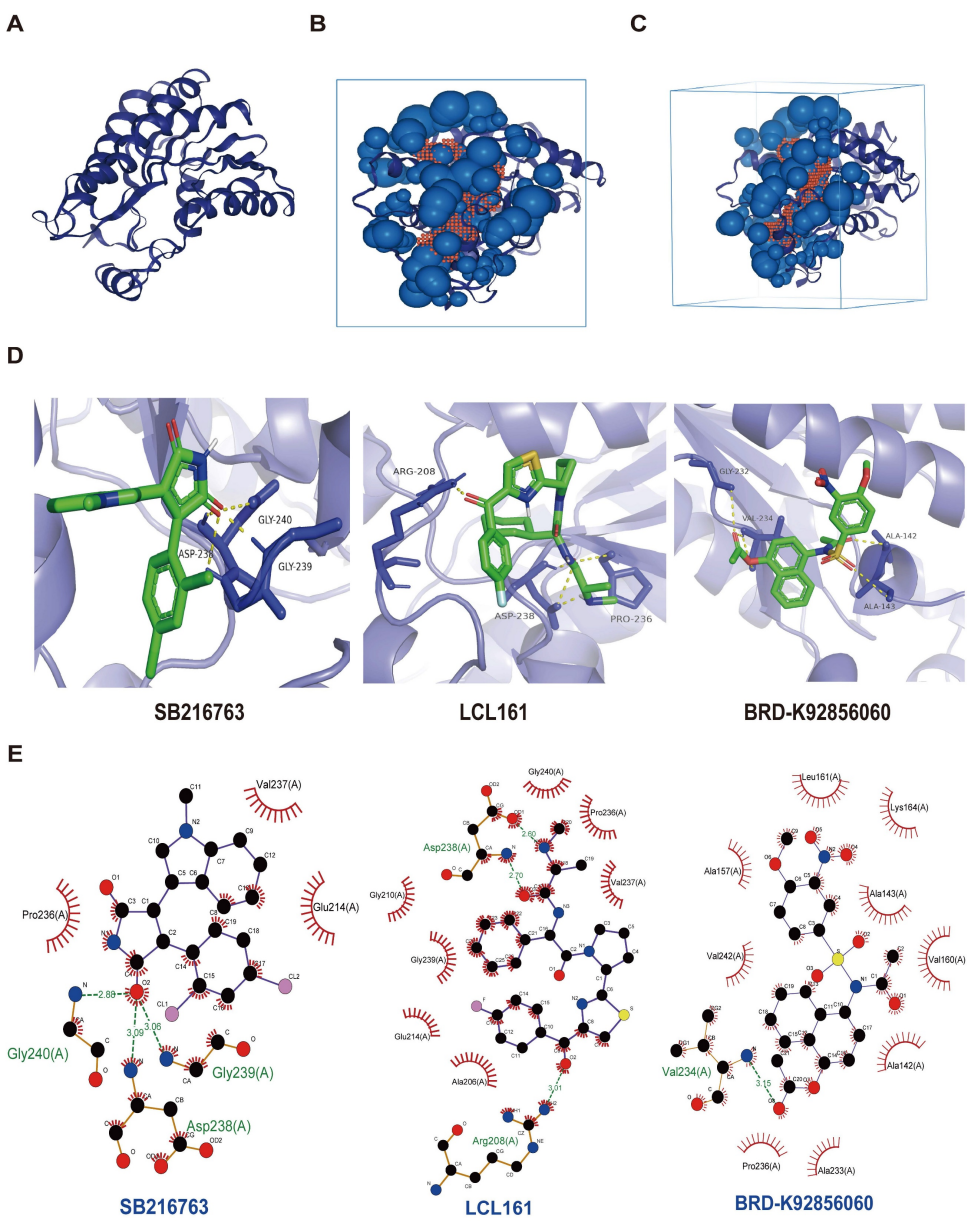
Screening sensitive drugs for PLAU protein. **A** Three-dimensional structure of the PLAU protein. **B-C** Predicting the potential binding sites and box of PLAU protein. **D** Analysis of the binding conformation of sensitive drugs to PLAU protein. **E** Visualization of the two-dimensional structure of drugs bound to PLAU protein.

Next, we screened the sensitive drugs for the *PLAU* and *GSDMC* proteins. We downloaded the IC50 values of drugs and gene expression levels of CRC cell lines from the GDSC2 and CTRPv2 databases. We subsequently analyzed the sensitivity of the *PLAU* and GDSMC proteins to drugs (Table S3). Scatter plots of the 8 drugs selected for the *PLAU* protein are shown in Fig. 6A, while scatter plots of the drugs selected for the *GSDMC* protein are shown in Fig. 6B. The top three drug structures were downloaded from the PubChem database (Fig. S5). Next, we obtained the spatial structures of the *PLAU* and *GSDMC* proteins from the PDB database (Fig. 7A and Fig. S6A). Using the CHECOM algorithm, we predicted the binding sites and boxes of the *PLAU* and *GSDMC* proteins (Fig. 7B-C and Fig. S6B-C). In addition, we utilized AutoDock software for molecular docking and obtained the free binding energies of the two proteins with the drugs (Table S4). Then, via PyMOL software, we visualized the binding of the selected drugs to the *PLAU* and *GSDMC* proteins (Fig. 7D and Fig. S6D). Finally, we used Ligplus software to visualize the two-dimensional interactions between the *PLAU* and *GSDMC* proteins and the small-molecule drugs (Fig. 7E and Fig. S6E).

### Super-enhancer activity regulates *PLAU* and *GSDMC* expression

**Figure 8:**
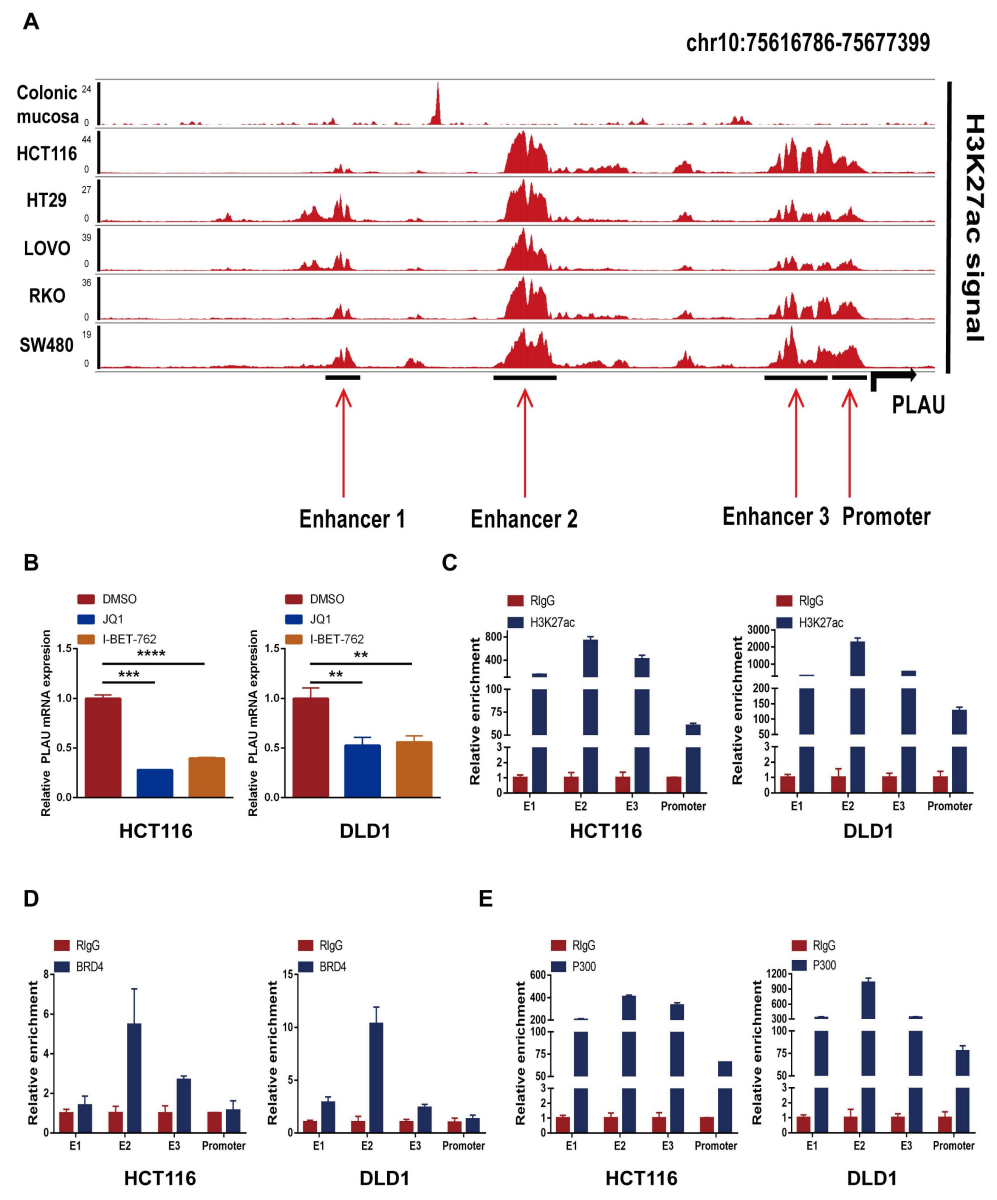
Effect of super-enhancer activity on *PLAU* expression. **A** ChIP-seq profiles of H3K27ac signal in the *PLAU* gene locus in different colorectal cancer cells. **B** The expression levels of *PLAU* in HCT116 and DLD1 cells treated with JQ1 and I-BET-762 were detected by qPCR. **C** The relative enrichment of H3K27ac in the *PLAU* gene locus by ChIP-qPCR. **D** The relative enrichment of BRD4 in the *PLAU* gene locus by ChIP-qPCR. **E** The relative enrichment of P300 in the *PLAU* gene locus by ChIP-qPCR. (Data are represented as mean ± SD, **p<0.01, ***p<0.001).

**Figure 9:**
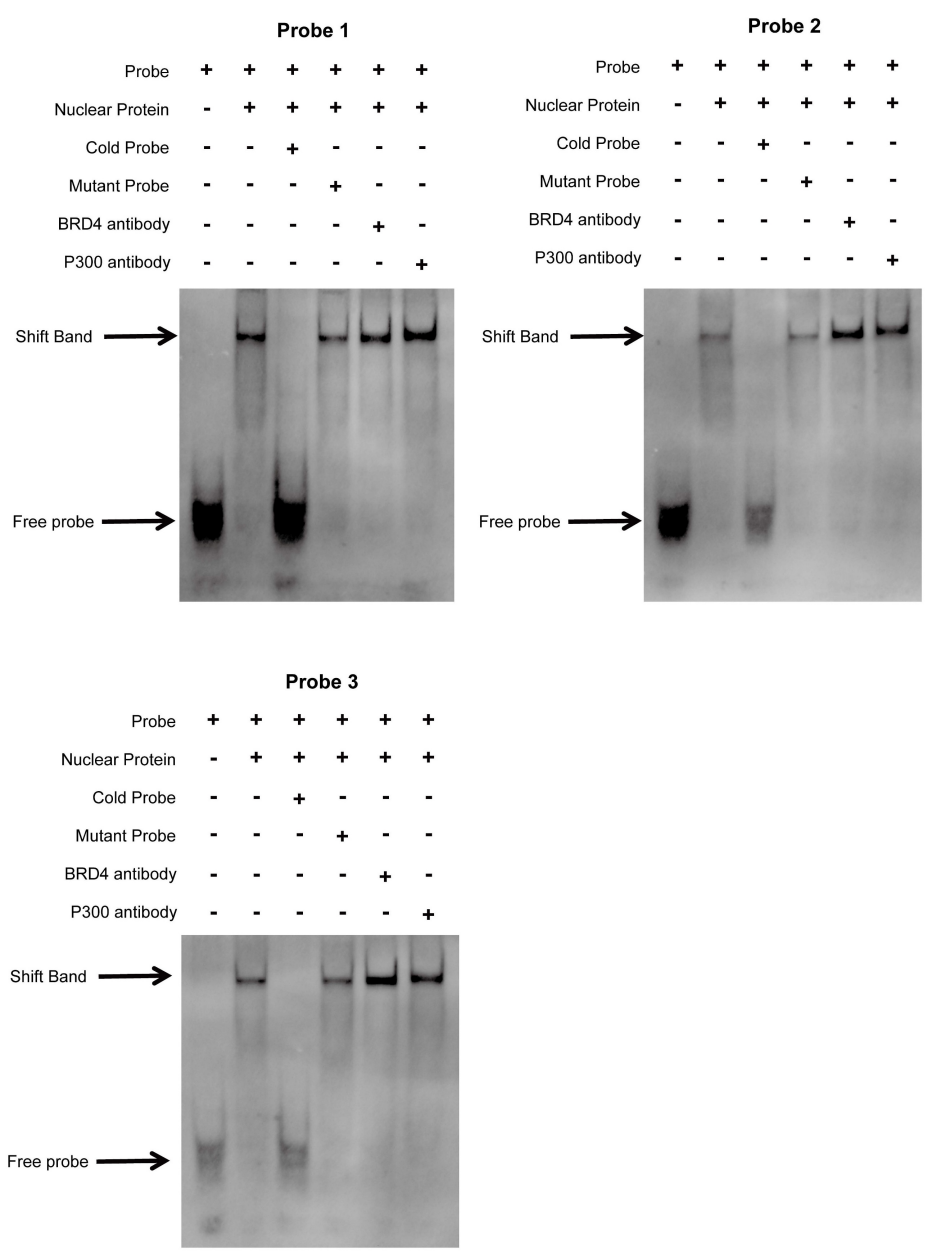
The interplay between BRD4/P300 and *PLAU* super-enhancers was validated using super-shift electrophoretic mobility shift assay (EMSA) in HCT116 cell.

We first validated the relative mRNA expression levels of *PLAU* and *GSDMC* in CRC using our clinical samples (Fig. S7A). Then we wondered whether the high expression of *PLAU* and *GSDMC* in CRC was up to the super-enhancer activity. We analyzed H3K27ac signals in the gene loci of *PLAU* and *GSDMC* in various COAD cells compared with those in normal colonic mucosa using ChIP-seq data obtained from the ENCODE database. We observed a greater H3K27ac signal in the *PLAU* gene loci than in the *GSDMC* gene loci (Fig. 8A and Fig. S7B). After treatment with the BRD4 inhibitors JQ1 and I-BET-762, which can block the formation of super-enhancers 38, the expression levels of *PLAU* and *GSDMC* decreased significantly in COAD cells (Fig. 8B and Fig. S7C). Through ChIP‒qPCR, we observed significant enrichment of H3K27ac and BRD4 expression at the super-enhancer sites of *PLAU* and *GSDMC* (Fig. 8C‒D and Fig. S7D). Interestingly, we also detected increased enrichment of the acetyltransferase P300 expression at these *PLAU* and *GSDMC* gene loci (Fig. 8E and Fig. S7E). Additionally, we conducted super-shift EMSAs of BRD4 and P300 to further validate our results (Fig. 9). We found that BRD4, especially P300, could bind to these *PLAU* super-enhancer sites.

## Discussion

ICIs have shown good clinical efficacy in CRC patients. However, owing to the heterogeneity of tumors, ICI treatment is known to be effective in only a small number of CRC patients. The existing biomarker MSI-H is considered as a good predictor of the response to ICIs. However, only 20% of CRC patients belong to the MSI-H type 39. Therefore, identifying new biomarkers to select patients who may respond well to ICIs is imperative.

Super-enhancers can drive increased levels of gene transcription 40 and play an important role in CRC 41. The super-enhancer is crucial for multiple tumor biological characteristics, including the immune response 42. Previous studies have demonstrated that the characteristics of the TME in tumors is closely related to the patient's response to immunotherapy 43, 44. Thus, understanding immune cell infiltration in the TME provides a powerful method for identifying CRC patients who are responsive to ICI therapy. In this study, we innovatively screened CRC-specific super-enhancer-related genes associated with immune infiltration by WGCNA. Notably, 83 genes in the turquoise module were positively correlated with immune infiltration scores, indicating that high expression of these genes may have a significant effect on the immunotherapy response of patients with colorectal cancer. Similarly, one recent study identified an immune-related module by WGCNA in lung cancer 45. Studying the expression of these 83 genes in colorectal cancer patients and their impact on prognosis is highly important for further screening of biomarkers for the diagnosis and prognosis of colorectal cancer. Therefore, a LASSO-Cox prognostic risk model was constructed on the basis of the TCGA-COAD cohort. By analyzing the relationship between risk model gene expression and the immune phenotype score (IPS), we found that *PLAU* and *GSDMC* have high predictive value for the efficacy of immunotherapy and may become new targets for CRC immunotherapy. The various components in the tumor microenvironment are closely related to immunotherapy efficacy 46. Therefore, we further investigated the correlation between the expression of *PLAU/GSDMC* and the immune microenvironment, which might explain why *PLAU/GSDMC* expression can predict immunotherapy efficacy.

Plasmin activator urokinase (*PLAU*), a protease involved in the conversion of plasminogen to plasmin 47, encodes a serine protease. Studies have shown that *PLAU* plays a key role in tumorigenesis and progression. By binding to their receptors, these proteases are converted into their active forms, enabling tumor cells to degrade surrounding extracellular matrix components. This process facilitates the invasion and migration of tumor cells 48. Additionally, *PLAU* is closely related to tumor diagnosis, treatment, and prognosis 49. Similarly, we identified a poor prognosis for patients with high *PLAU* expression in CRC, suggesting that *PLAU* could serve as a biomarker for predicting the prognosis of patients with CRC. Previous studies have shown that the overexpression of *PLAU* is associated with the immunosuppressive tumor microenvironment in pancreatic ductal adenocarcinoma. The overexpression of *PLAU* was more closely associated with PD-L1 and PD-L2 but had a weaker correlation with antitumor immunity 50. Our research revealed a significant positive correlation between *PLAU* expression and the tumor microenvironment in CRC. More importantly, *PLAU* expression was positively correlated with the IPS, which is an effective means of predicting the ICI response 31. CRCs with mismatch repair defects (dMMR) or severely mutated tumors with MSI-H can be effectively treated with ICIs 51. Interestingly, we also found a positive correlation between *PLAU* expression and MSI scores in CRC. Furthermore, we found that *PLAU* had a better ability to predict immunotherapy efficacy than some existing biomarkers, such as MSI and TMB, which have become reliable biomarkers for predicting the efficacy of ICIs in certain cancers 52. Unexpectedly, we found that *PLAU* expression might affect the function of cytotoxic T cells but not their abundance. Similarly, *PLAU* is negatively related to CD8^+^ T-cell invasion of head and neck squamous cell carcinomas 53. These data indicated that high expression of *PLAU* could aid tumor cells in evading the immune system by impairing the function of T cells. This finding may be helpful to explain immunotherapy resistance. These results suggest that *PLAU* may serve not only as an effective biomarker for predicting the efficacy of immunotherapy but also as a potential new target for immunotherapy in CRC.

*GSDMC* is a member of the GSDM family that has been extensively studied in recent years for its role in promoting cell pyroptosis^54^. *GSDMC* is highly expressed in CRC, and the proliferation of colorectal cells is reduced after *GSDMC* is silenced 55. In addition, in many tumor types, patients with high *GSDMC* expression have a poor prognosis 56. PD-1-mediated *GSDMC* expression converts apoptosis to necroptosis in cancer cells 57. *GSDMC* was found to be valuable in predicting the outcome and immunotherapy response of patients with pancreatic adenocarcinoma 58. In addition, *GSDMC* expression was associated with an immune-hot tumor microenvironment 59. Similarly, our study revealed that *GSDMC* was positively correlated with immune scores, immune cell infiltration, and the expression of immune checkpoint genes. These findings indicate that* GSDMC* may affect the immunotherapy efficacy of colorectal cancer by altering immune cells, stromal cells, and other components in the immune microenvironment of CRC. Therefore, when immunotherapy is applied, CRC patients with high *GSDMC* expression are likely to have better outcomes. Indeed, we found that *GSDMC* expression was positively correlated with IPS and MSI scores, suggesting that *GSDMC*, like *PLAU*, could serve as a biomarker for predicting CRC immunotherapy efficacy. Notably, we found that high expression of *GSDMC* had an impact on the function of cytotoxic T cells. Therefore, we speculate that *GSDMC* expression might become a new biomarker for immunotherapy.

Additionally, we elucidated the reasons why *PLAU* and *GSDMC* can predict the efficacy of immunotherapy. First, there is growing evidence that immune cells in the tumor microenvironment play a key role in tumorigenesis and progression 10. Research has shown that tumor-associated macrophages (TAMs) can express high levels of anti-inflammatory factors to promote an inhibitory immune microenvironment and affect antitumor immunity 60. TAMs can also inhibit the recruitment and activation of T cells, thereby exacerbating immune suppression 61. Cancer-associated fibroblasts (CAFs) can produce inhibitory cytokines, suppress the accumulation of T cells near tumors, and affect the efficacy of immunotherapy 62. In addition, a high abundance of Treg cells in the TME is associated with immune suppression and a poor prognosis 63, 64. Our research revealed a significant positive correlation between the expression of *PLAU*/*GSDMC* and infiltrating immune cells in CRC. This implies that the higher the expression levels of these two genes are, the greater the number of immune cells in the tumor microenvironment. In line with our findings, the overexpression of *PLAU* is positively correlated with the infiltration of various immune cells in pancreatic cancer 65. *PLAU* is also strongly positively correlated with tumor-infiltrating lymphocytes in lung adenocarcinoma 66. The expression of *PLAU* may promote an immune-hot tumor microenvironment. Therefore, the use of immune checkpoint inhibitors results in better therapeutic effects. Second, we were surprised to observe that the expression level of *PLAU*/*GSDMC* was positively correlated with immune checkpoint genes. In other words, when the expression of these two genes increases, the expression of immune checkpoint genes also increases, increasing the effectiveness of treatment with immune checkpoint inhibitors. Currently, many patients who are theoretically suitable for treatment with ICIs are resistant to PD-1 or PD-L1 inhibitors due to various factors. For example, deletion of the β-2-microglobulin heterodimer may affect the antigen presentation of MHC-I and induce resistance to ICIs 67. Interestingly, studies have demonstrated the costimulatory effect of LCL161 under conditions in which TAC-T cells are stimulated with antigen alone, and increased T-cell survival and proliferation have been observed 68. Considering the correlation between *PLAU*/*GSDMC* expression and immune cell infiltration, as well as PD-1/PD-L1/CTLA4 expression, we hypothesized that better immunotherapy efficacy could be achieved by targeting PLAU/GSDMC proteins in combination with ICIs. Therefore, we utilized drug sensitivity analysis and virtual molecular docking to screen for drugs that are able to target PLAU/GSDMC proteins. Overall, our study indicated that targeting PLAU/GSDMC may offer a potential new strategy for the immunotherapy of CRC.

Super-enhancer-driven genes have potential as biomarkers and therapeutic targets for cancers 69. In our study, we observed high H3K27ac signal at the *PLAU* gene locus. In addition, our in vitro experiments indicated that the marker-H3K27ac, the reader-BRD4, and the creater-P300 of super-enhancers were all highly enriched at the enhancer sites of *PLAU*. In sum, our results revealed that super-enhancer activity was responsible for the expression of *PLAU* in CRC. Therefore, our study revealed a link between the super-enhancer and CRC. Interestingly, recent studies have reported the impact of immune infiltration super-enhancer regulatory genes on immunotherapy efficacy in gastrointestinal tumors 70. Similarly, our latest study revealed that super-enhancer-induced LINC00862 could serve as a biomarker for predicting immunotherapy efficacy 71. Overall, we confirmed the presence of super-enhancers and the influence of super-enhancer activity on *PLAU* expression.

At present, reliable biomarkers for the immunotherapy of colorectal cancer, such as dMMR/MSI-H, can only benefit approximately 15% of colorectal cancer patients. Therefore, the identification of similar biomarkers can aid in the accurate selection of patients who may benefit from immunotherapy, thereby avoiding ineffective treatment. In addition, by screening reliable biomarkers, the disease development trend and prognosis of colorectal cancer patients can be understood in advance, and treatment plans can be optimized. This study investigated the correlation between the expression of *PLAU* or *GSDMC* and the tumor microenvironment of colorectal cancer and revealed that high expression of *PLAU* and *GSDMC* maybe regulate the function of CTL cells. Therefore, our study provides potential new biomarkers for predicting the efficacy of immunotherapy and possibly new targets for immunotherapy in CRC. Besides, on the basis of these newly discovered biomarkers, this study can lead to the development of new targeted drugs and provide new options for patients with colorectal cancer. However, data on immunotherapy for colorectal cancer in existing databases are lacking, and the correlation between *PLAU* and *GSDMC* expression and the prognosis of patients with colorectal cancer treated with immunotherapy is currently unclear. Therefore, we analyzed the impact of *PLAU* and *GSDMC* expression on the prognosis of melanoma patients treated with ICIs. In the future, we will continue to collect relevant samples of patients with colorectal cancer treated with immunotherapy and further study the correlation between *PLAU* and *GSDMC* expression and the prognosis of colorectal cancer patients treated with ICIs. As found in Ke et al.'s latest research, the expression of PD-1 and CD69 on effector memory CD8+ T cells in the blood can predict the presence of tertiary lymphoid structures (TLSs) in the macroscopic environment of CRC and serve as biomarkers for stratifying CRC patients for immunotherapy 72. In the coming years, through the application of large-scale biological data analysis and single-cell sequencing, a more comprehensive understanding of the tumor microenvironment of colorectal cancer can be achieved, facilitating the exploration of more biomarkers that reflect the tumor immune status and predict the immunotherapy response. In addition, by combining existing biomarkers such as MSI, TMB, and PD-L1, with newly discovered biomarkers, the accuracy of predicting immunotherapy efficacy and patient stratification for colorectal cancer can be improved.

Our research has several limitations. First, few colorectal cancer patients rely solely on immunotherapy in clinical practice, and the sample collection requires a longer period of time. Therefore, we were unable to analyze the relationship between *PLAU* and *GSDMC* expression and the prognosis of colorectal cancer patients treated with ICIs. In addition, owing to the limited collection of IC50 data for drugs in existing databases, it is not possible to comprehensively evaluate the sensitivity of drugs. And the complex relationship between diseases and drugs cannot be fully considered in the screening process, resulting in possible biases in the screening results. Furthermore, the construction period of animal models is relatively long, and the in vitro reactions of drugs differ greatly from the actual situation of human trials. Therefore, this study has not yet conducted further research on drug sensitivity for PLAU and GSDMC proteins. Finally, in the initial stage of this study, the correlations between *PLAU* and *GSDMC* expression and the tumor microenvironment of patients with colorectal cancer were analyzed using bioinformatics methods. Further experiments are needed to clarify the specific regulatory mechanisms involved.

## Conclusion

Collectively, our study identifies super-enhancer-based biomarkers for predicting survival and immunotherapy efficacy in CRC. The six super-enhancer-related signatures could act as a potential prognostic biomarker for CRC. *PLAU* and *GSDMC* expression had high predictive value for immunotherapy efficacy.

## Supplementary Material

Supplementary figures and tables.
