# Supplementary material for: Identification of super-enhancer-based biomarkers for predicting survival and immunotherapy efficacy in colorectal cancer
Source: J Cancer. 2026 Jan 1;17(2):338–58. doi: 10.7150/jca.119265 (PMC12825423; doi:10.7150/jca.119265)
Supplement: Supplementary file 1 — Supplementary figures and tables. [file jcav17p0338s1.pdf]

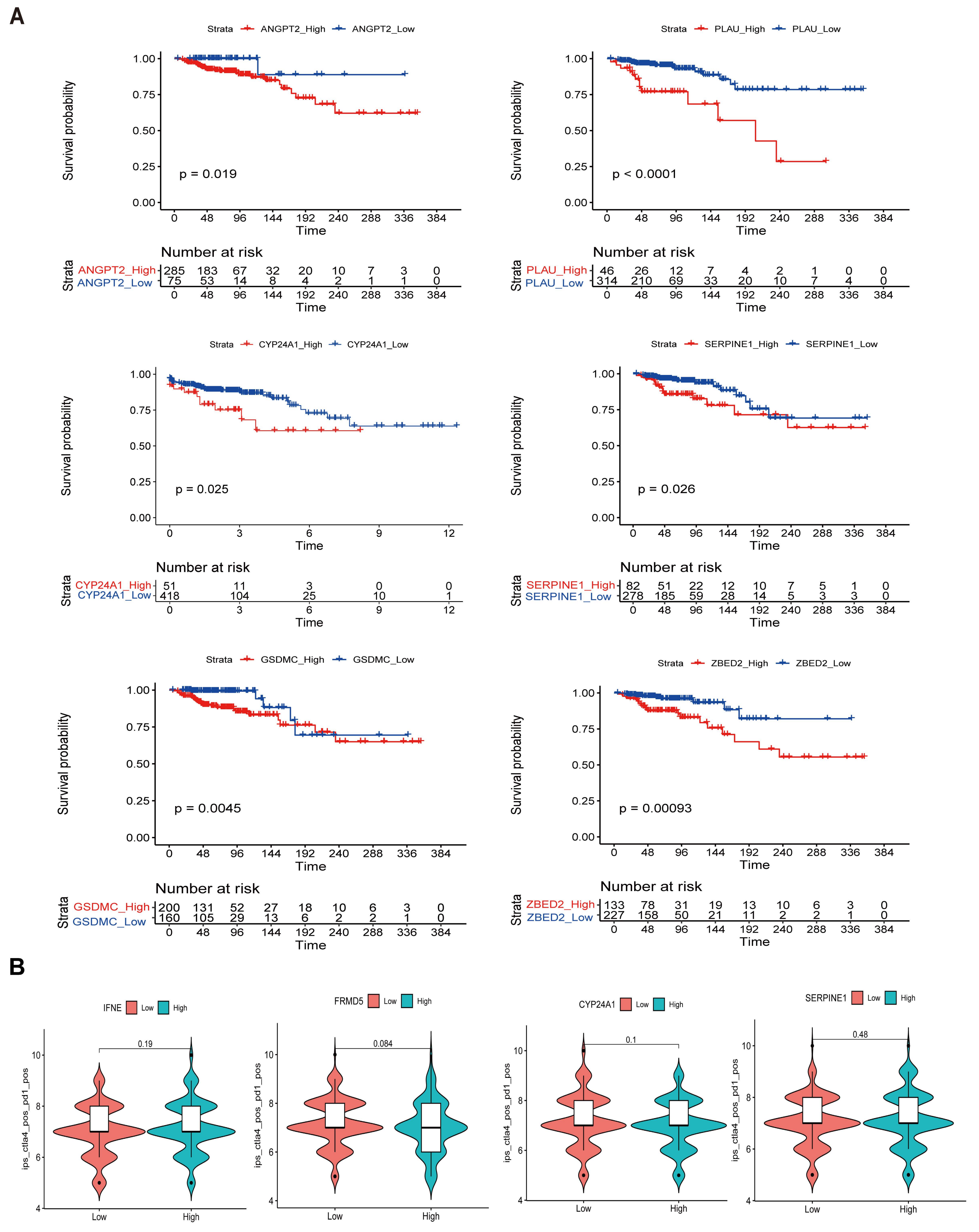

Fig. S1. A. K-M curves showing the prognostic value of genes in the TCGA database. B. Relationship between gene expression and IPS scores.

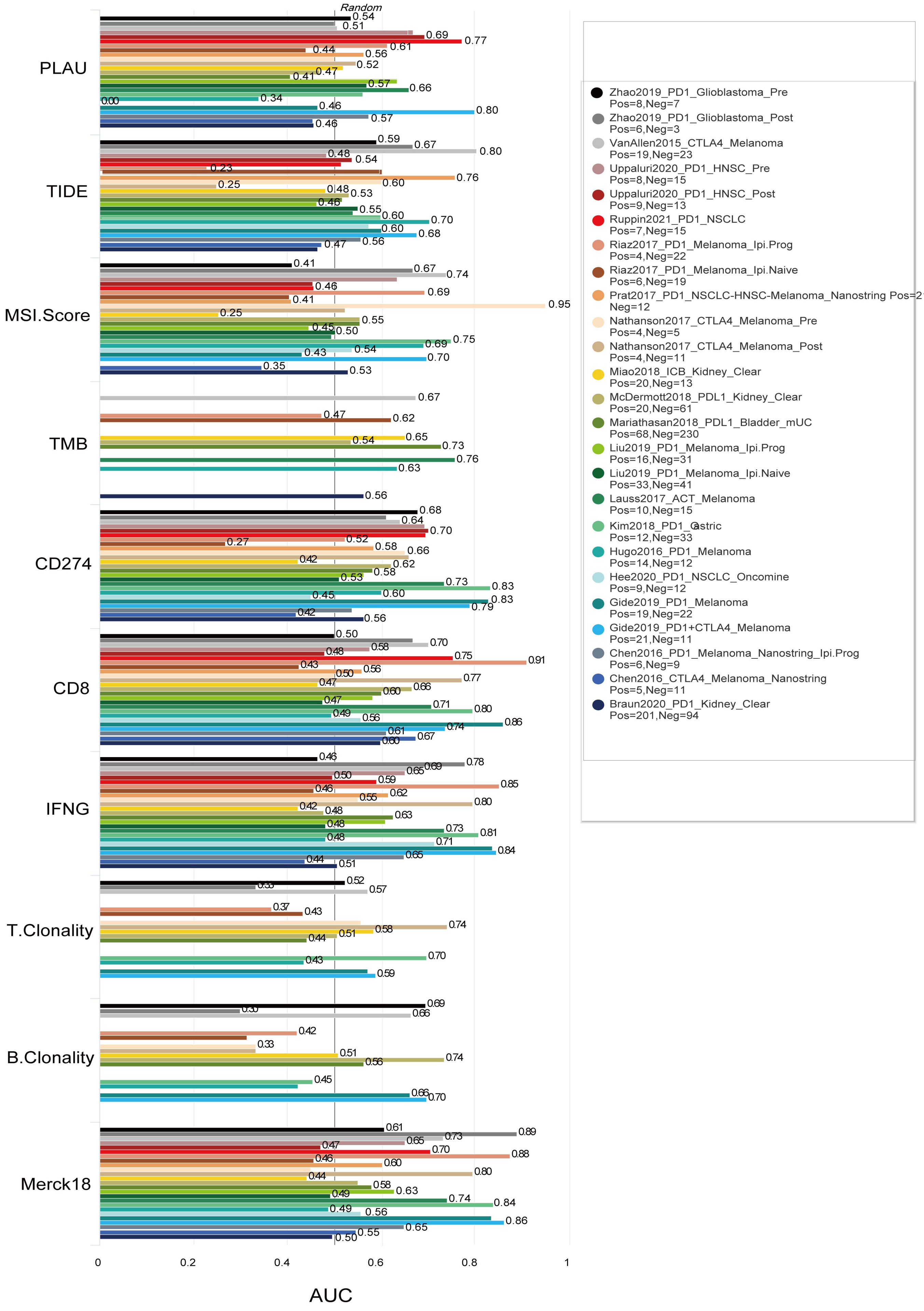

Fig. S2. The predictive capability of PLAU for immunotherapy efficacy in multiple human immunotherapy cohorts. AUC values >0.5 means that the algorithm outperformed random.

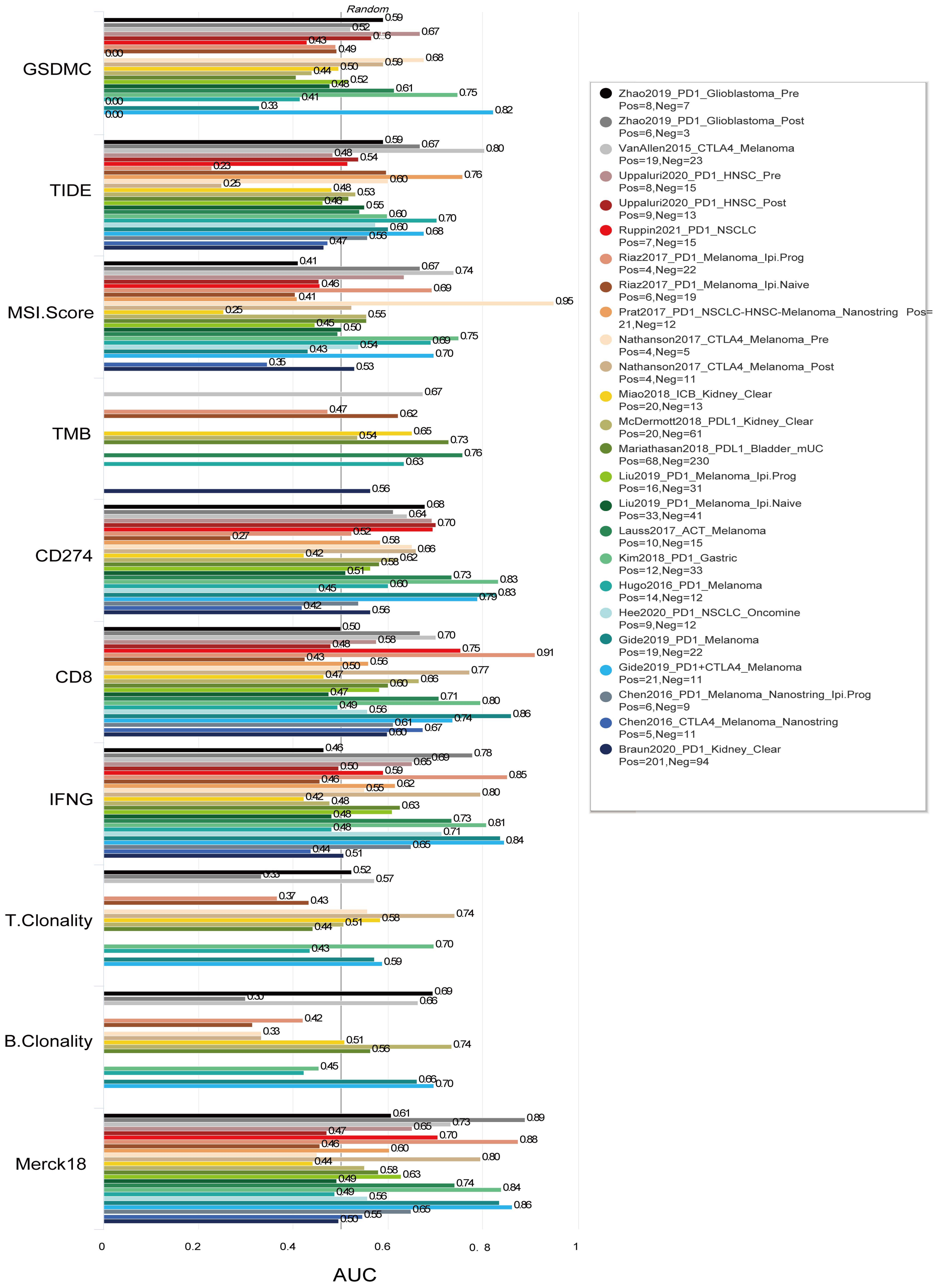

Fig. S3. The predictive capability of GSDMC for immunotherapy efficacy in multiple human immunotherapy cohorts. AUC values >0.5 means that the algorithm outperformed random.

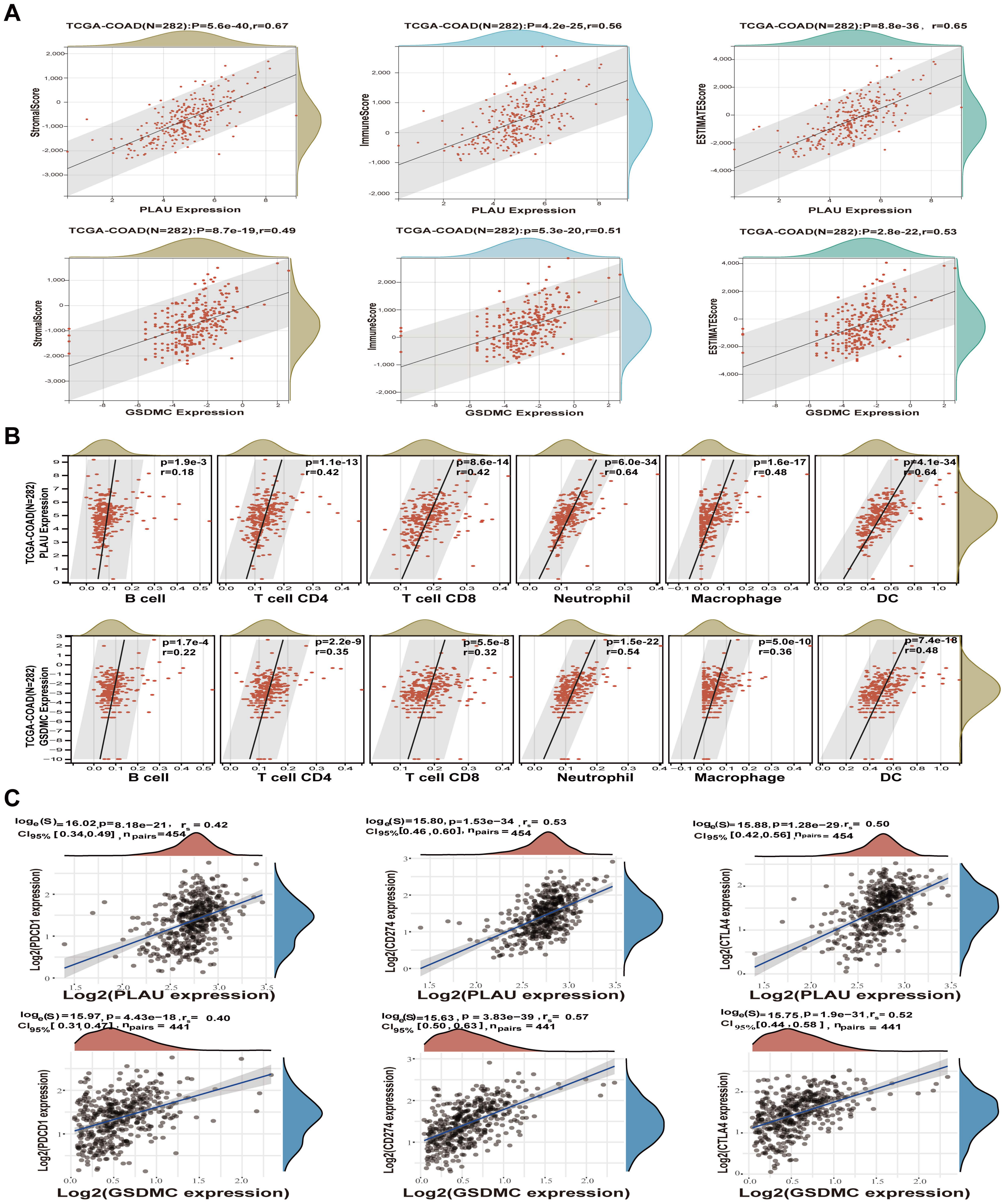

Fig. S4. The correlation between PLAU and GSDMC and tumor microenvironment. A. Spearman correlation between PLAU and GSDMC and immune infiltration scores in COAD. B. Spearman correlation of PLAU and GSDMC expression with immune cell infiltration in COAD. C. Spearman correlation of PLAU and GSDMC expression with immune checkpoint genes in COAD.

A

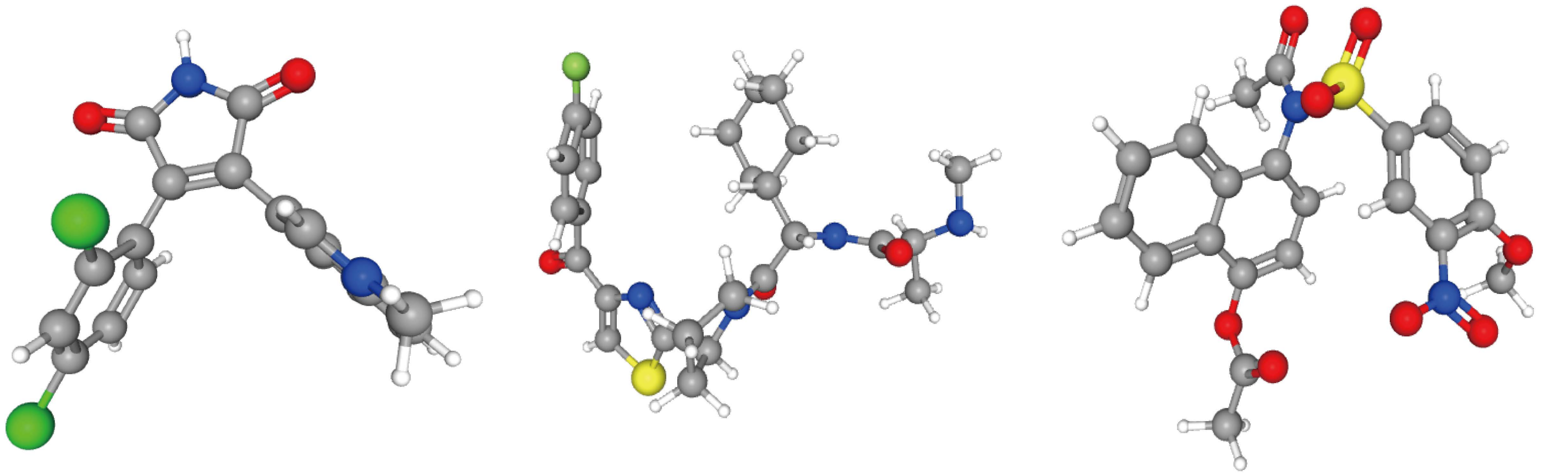

SB216763 LCL161 BRD-K92856060

B

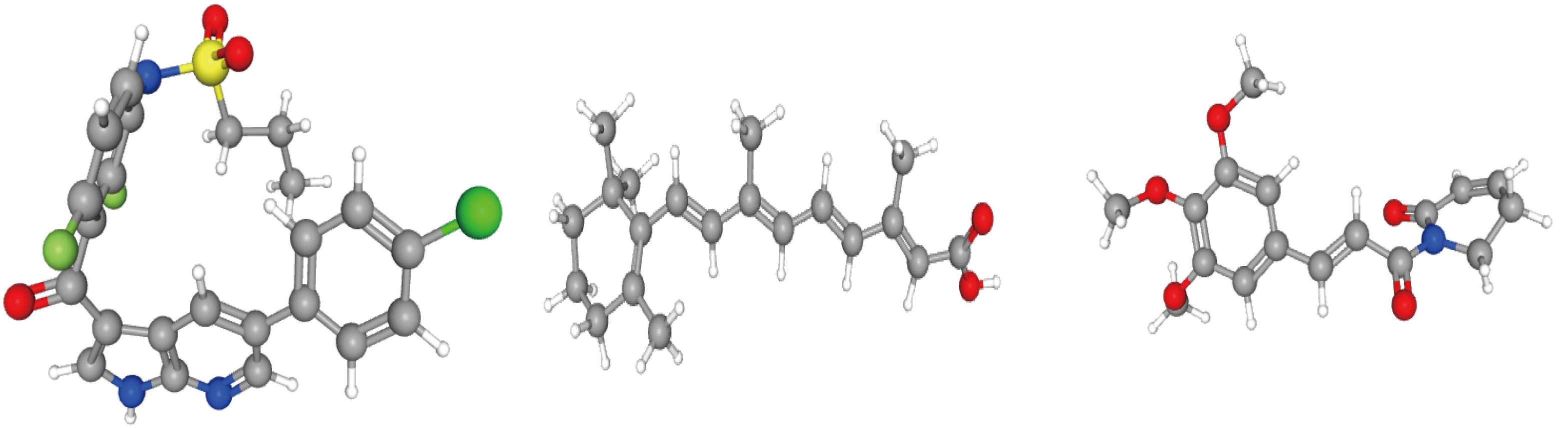

PLX-4032 Tretinoin Piperlongumine

Fig. S5. Three-dimensional structure of small molecule drugs. A. Three drug structures with revelance to PLAU protein. B. Three drug structures with relevance to GSDMC protein.

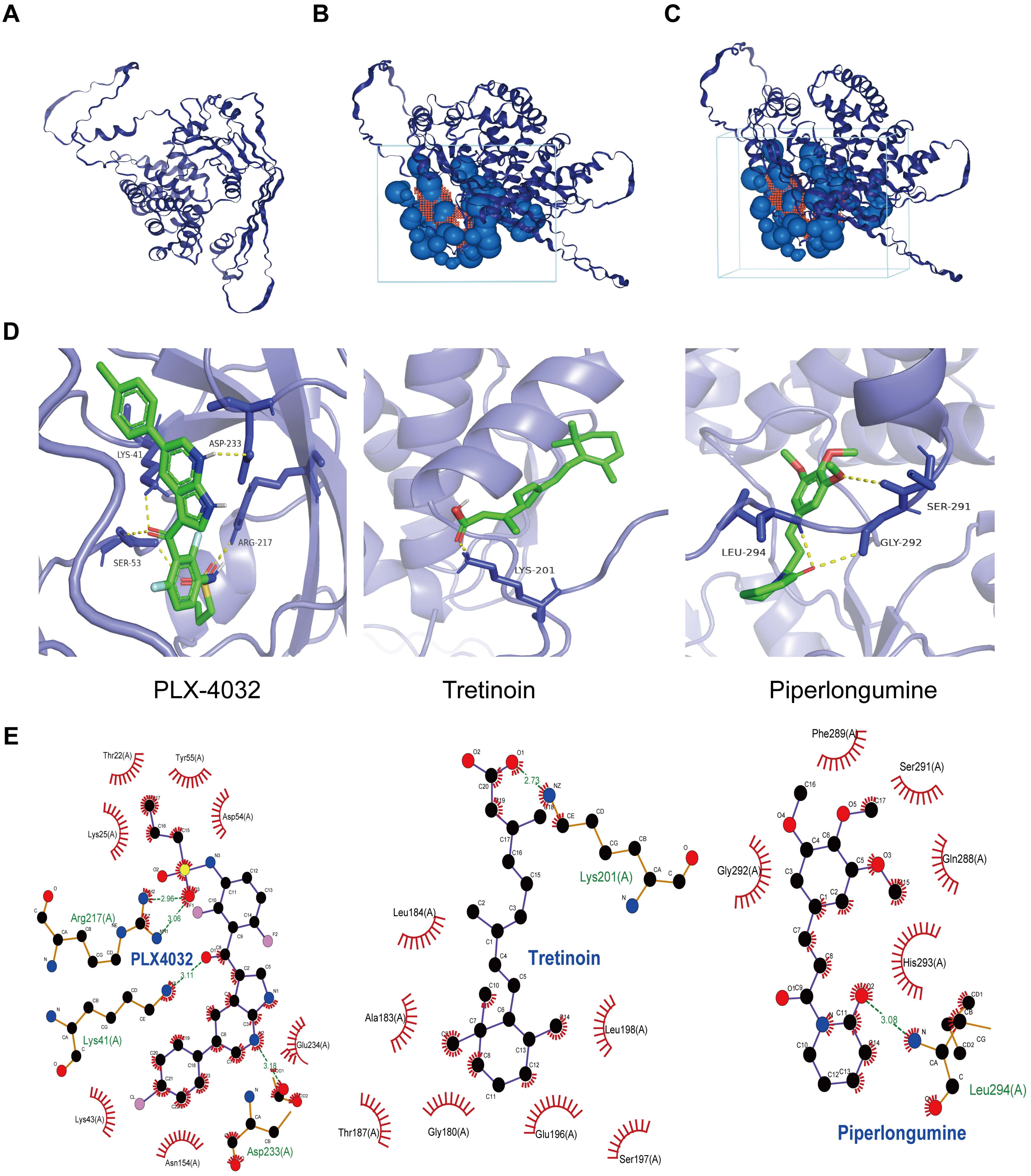

Fig. S6. Screening sensitive drugs for GSDMC protein. A. Three-dimensional structure of the GSDMC protein. B-C. Predicting the potential binding sites and box on GSDMC protein. D. Analysis of the binding conformation of sensitive drugs to GSDMC protein. E. Visualization of the two-dimensional structure of drugs bound to GSDMC protein.

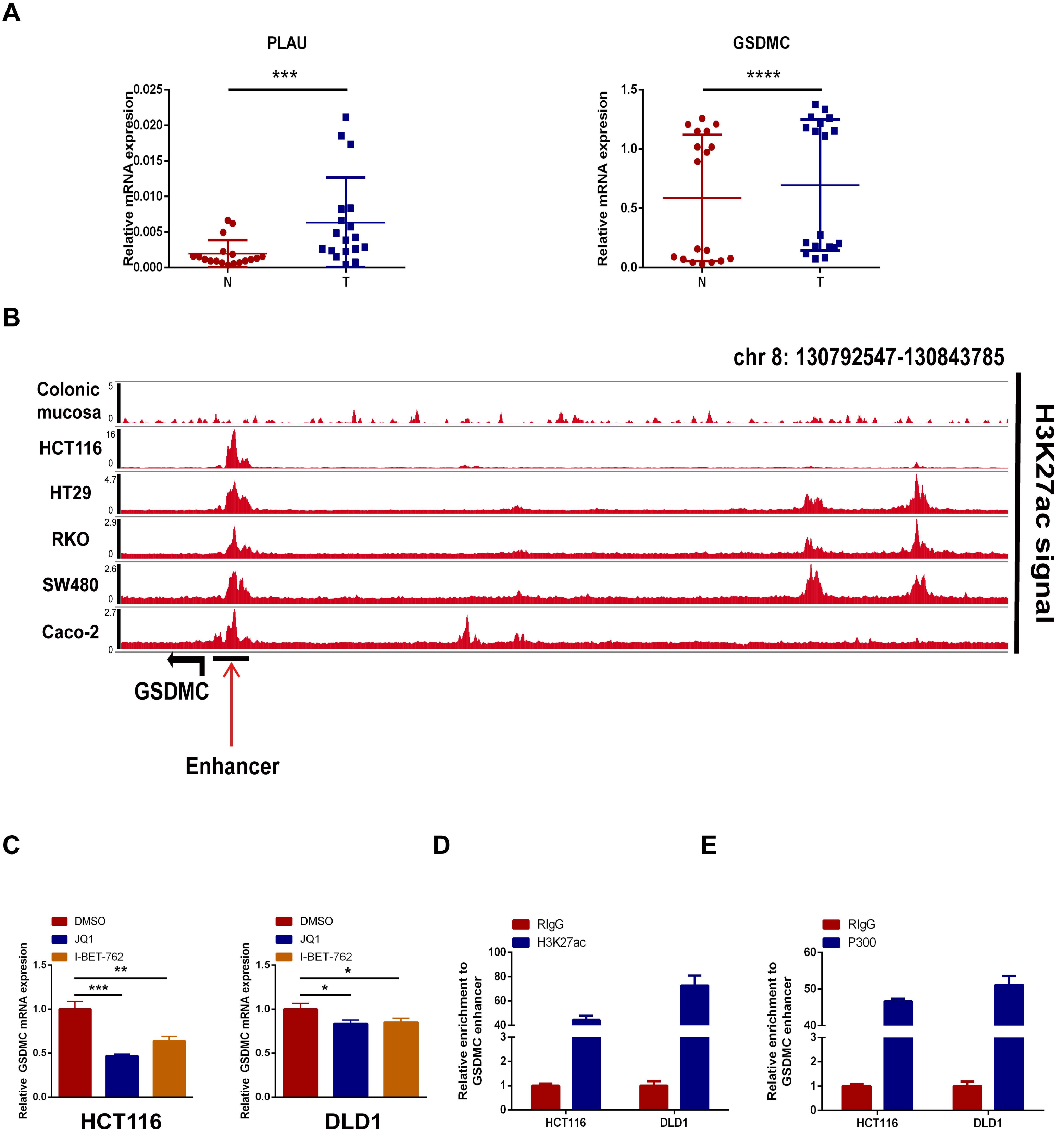

Fig. S7. A. The PLAU and GSDMC mRNA levels are upregulated in colorectal cancer tissues. B. ChIP-seq profiles of H3K27ac signal in the GSDMC gene locus in different colorectal cancer cells. C. The expression levels of GSDMC in HCT116 and DLD1 cells treated with JQ1 and I-BET-762 were detected by qPCR. D. The relative enrichment of H3K27ac in the GSDMC gene locus by ChIP-qPCR. E. The relative enrichment of P300 in the GSDMC gene locus by ChIP-qPCR.

Table S1. Optimal cutpoint values for survival analysis in TCGA data

Gene	Cutpoint	Statistic
**ANGPT2**	4.9976	1.920288
**PLAU**	22.6167	4.614342
**ZBED2**	0.4577	3.527775
**GSDMC**	0.3892	2.780785
**SERPINE1**	13.1105	2.318292
**CYP24A1**	0.0122	2.193734

Table S2. Risk genes through LASSO-COX regression analysis

Gene	Coef
**FRMD5**	0.216124258026923
**SERPINE1**	0.380973652346092
**IFNE**	0.0324982140801412
**GSDMC**	-0.100682905697296
**PLAU**	-0.426532126648938
**CYP24A1**	-0.0722131730892283

Table S3. Predicted sensitive drugs for PLAU and GSDMC protein

Gene	Drug	correlation	P value
**PLAU**	LCL161	-0.384101575864601	0.0100508963427865
**PLAU**	BRD-K92856060	-0.354598006593615	0.0181894618432729
**PLAU**	SB216763	-0.345849056176333	0.0214749590774912
**PLAU**	pifithrin-alpha	-0.345438430151654	0.0216406558417136
**PLAU**	OSI-027	-0.322293723193538	0.0328749889192166
**PLAU**	BRD4132	-0.303956960192454	0.0448650047546461
**PLAU**	CZC24832	-0.314594126928421	0.0375401854291833
**PLAU**	KH-CB19	-0.298226223304054	0.0492687879305438
**GSDMC**	PLX-4032	-0.604406556849717	1.39463661374655e-05
**GSDMC**	compound 1B	-0.44997122838213	0.00217956637794985
**GSDMC**	tretinoin	-0.392861597977969	0.0083414534268593
**GSDMC**	ciclosporin	-0.390148362319898	0.00884187985649159
**GSDMC**	navitoclax:piperlongumine (1:1 mol/mol)	-0.348109390957182	0.02058180123836
**GSDMC**	tretinoin:navitoclax (4:1 mol/mol)	-0.329892280309851	0.0287506472488793
**GSDMC**	navitoclax:pluripotin (1:1 mol/mol)	-0.324118006689932	0.0318427945125299
**GSDMC**	navitoclax:MST-312 (1:1 mol/mol)	-0.313115279046431	0.0384955684563447
**GSDMC**	tretinoin:carboplatin (2:1 mol/mol)	-0.302258813280389	0.0461352281859266

Table S4. Energy values docking simulation of PLAU and GSDMC with drugs

Gene	Drug	Estimated Free Energy of Binding
**PLAU**	SB216763	-4.15 kcal/mol
**PLAU**	LCL161	-5.59 kcal/mol
**PLAU**	BRD-K92856060	- 6.17kcal/mol
**GSDMC**	PLX-4032	-3.57 kcal/mol
**GSDMC**	Tretinoin	-4.08 kcal/mol
**GSDMC**	Piperlongumine	-2.99 kcal/mol
